# Micronuclei in Bone Marrow and Liver in relation to Hepatic Metabolism and Antioxidant Response due to Coexposure to Chloroform, Dichloromethane, and Toluene in the Rat Model

**DOI:** 10.1155/2014/425070

**Published:** 2014-05-14

**Authors:** Javier Belmont-Díaz, Ana Paulina López-Gordillo, Eunice Molina Garduño, Luis Serrano-García, Elvia Coballase-Urrutia, Noemí Cárdenas-Rodríguez, Omar Arellano-Aguilar, Regina D. Montero-Montoya

**Affiliations:** ^1^Departamento de Medicina Genómica y Toxicología Ambiental, Instituto de Investigaciones Biomédicas, Universidad Nacional Autónoma de México, Apartado Postal 70228, 04510 México, DF, Mexico; ^2^Departamento de Bioquímica, Instituto Nacional de Cardiología Ignacio Chávez, Juan Badiano No. 1, Tlalpan, 14080 México, DF, Mexico; ^3^Laboratorio de Neuroquímica, Instituto Nacional de Pediatría, Insurgentes Sur 3700-C, 04530 México, DF, Mexico

## Abstract

Genotoxicity in cells may occur in different ways, direct interaction, production of electrophilic metabolites, and secondary genotoxicity via oxidative stress. Chloroform, dichloromethane, and toluene are primarily metabolized in liver by CYP2E1, producing reactive electrophilic metabolites, and may also produce oxidative stress via the uncoupled CYP2E1 catalytic cycle. Additionally, GSTT1 also participates in dichloromethane activation. Despite the oxidative metabolism of these compounds and the production of oxidative adducts, their genotoxicity in the bone marrow micronucleus test is unclear. The objective of this work was to analyze whether the oxidative metabolism induced by the coexposure to these compounds would account for increased micronucleus frequency. We used an approach including the analysis of phase I, phase II, and antioxidant enzymes, oxidative stress biomarkers, and micronuclei in bone marrow (MNPCE) and hepatocytes (MNHEP). Rats were administered different doses of an artificial mixture of CLF/DCM/TOL, under two regimes. After one administration MNPCE frequency increased in correlation with induced GSTT1 activity and no oxidative stress occurred. Conversely, after three-day treatments oxidative stress was observed, without genotoxicity. The effects observed indicate that MNPCE by the coexposure to these VOCs could be increased via inducing the activity of metabolism enzymes.

## 1. Introduction

Genotoxic compounds are known to exert their effects on DNA either in a direct way or through their metabolites after going through an enzymatic transformation. Some compounds, however, have been described as being genotoxic via alternative pathways, like the production of ROS.

That is the case of DCM, which is primarily metabolized by CYP2E1 [1] into carbon monoxide which irreversibly binds to hemoglobin. However, under circumstances where CYP2E1 activity is inhibited or saturated it can also be metabolized into S-chloromethylglutathione by GSTT1 [2] and form DNA adducts [3].

CLF is another compound of this kind, capable of increasing malondialdehyde deoxyguanosine (M1dG) adducts and lipid peroxidation in HepG2 cells [4] via a CYP2E1 mediated oxidation where phosgene and, under anoxic conditions, dichloromethyl are produced [5], both being highly reactive electrophiles, able to form irreversible covalent bonds with biomolecules like lipids, proteins, and reduced glutathione (GSH) and to induce oxidative stress [6, 7].

TOL biotransformation also occurs through CYP2E1 [8], and during its metabolism in rat liver and brain an increased level of reactive oxygen species (ROS) is produced [9]. Its effect on a coexposure with benzene resulted in increased genotoxicity of benzene coupled with reduced glutathione [10].

In spite of the described oxidative metabolism of chloroform, dichloromethane, and toluene, and the formation of oxidative adducts produced by them, their genotoxicity in the micronucleus test, as many studies demonstrate, is not clear (Table 1). We became interested in this subject due to the fact that these three compounds have been detected in rivers polluted with industrial waste affecting animal populations; due to their high volatility these pollutants can be spread through the air and contaminate large areas, constituting a risk of exposure for every living being around. Particularly, these polluted rivers are very close to inhabited areas of agricultural activity [11, 12].

Due to the fact that cytochrome P-450-isoform CYP2E1 (CYP2E1) is mainly responsible for the oxidative metabolism of these VOCs [13, 14] and that this enzyme is known to be capable of inducing reactive oxygen species (ROS) [15, 16] which increase when the enzyme is induced, it appeared possible that under a coexposure to the three compounds clastogenicity could occur that might be detected in the form of micronuclei.

A pilot study was conducted where each compound was administered for three days in doses equivalent to the 10% of the LD50 in rats to analyze whether they would induce oxidative stress and, consequently, micronuclei in bone marrow polychromatic erythrocytes (MNPCE). No genotoxic effects were found with any of the compounds, and CLF and TOL induced CYP2E1 activity. Oxidative stress, measured by the levels of GSH in liver homogenate, was not detected under any treatment (Table 1). These results were used to design a greater study to analyze more parameters on the oxidative response and using different concentrations of the combined compounds. Furthermore, we added the analysis of micronuclei in hepatocytes due to the fact that the metabolism of the three compounds occurs mainly in the liver, irrespective of the route of administration [1, 6, 17] and the more likely target of genotoxicity would be this organ.

Therefore, in the present study a rat model was used to analyze the hepatic xenobiotic metabolism response (P-450 levels, CYP2E1, GST, and GSTT1 activities), the antioxidant response (antioxidant enzymes activity, GSH/GSSG, and TBARS), and whether there would be a relationship of these responses with the genotoxic damage in liver or in bone marrow that could be produced by the coexposure to the three compounds.

## 2. Materials and Methods

### 2.1. Reagents

HPLC grade chloroform (CAS: 67-66-3), dichloromethane (CAS: 75-09-2), and toluene (CAS: 108-88-3) were purchased from Honeywell Burdick & Jackson (Muskegon, MI, USA); protein assay dye reagent and acetylacetone were purchased from Bio-Rad (CA, USA). All other reagents were purchased from Sigma-Aldrich.

### 2.2. Maintenance of Animals

Three-week-old male Wistar rats (89.5 g ± 14.5) were maintained under controlled temperature 20 ± 2°C and 12 h light/dark cycles for one week prior to the treatment. Animals were fed with a commercial rat chow diet and water* ad libitum*. This study was conducted in compliance with the Mexican Regulations of Good Laboratory Practice (NOM-062-ZOO-1999) and was approved by the ethics committee of our institute.

### 2.3. Route of Exposure

Toluene, chloroform, and methylene chloride, regardless of the route of exposure, are distributed widely throughout the body (toluene in liver and brain) and their metabolism occurs mainly in the liver [1, 6, 17–20]. Furthermore, CLF was found to exhibit similar clastogenic effect in the rat chromosomal aberrations test administered either by oral or i.p. route [21], and TOL exerts its neurological effects when either orally or intraperitoneally administered [17]. By using the i.p. route, we made the toxicants enter the portal circulation and be metabolized in the liver [22], their main organ of metabolism, in order to obtain a maximal response for the production of genotoxicity if there was one. The i.p. route also ensured the internal dose of the three compounds.

### 2.4. Pilot Study

The genotoxic potential, induction of P-450, and reduced glutathione levels for separated treatments with CLF, DCM, or TOL, at i.p. doses of 2.5, 2.6, and 8.1 mmol/kg/day/3 days, respectively (corresponding to 1/10 LD50 of each compound, based on the LD50 reported in the Merck Index), were tested in rats, five animals/compound, in order to approximate the doses that would be used in the mixture.

Due to the wide variability of the environmental levels reported, our protocol was based on LD50 in order to obtain the maximum biological response in the shortest time (according to MNPCE protocols, by Krishna and Hayashi [32]) and to ensure that all components of the mixture would behave with approximately the same toxic potential; for this reason, the doses used are superior to the levels reported in the polluted rivers. However, our doses approximate to other exposure scenarios; for example, toluene is inhaled by some people for its euphoric properties and their exposure approximate 1000–10000 ppm which corresponds to an absorption of 0.2–2 mmol/kg/hr [19]. In relation to occupational exposures, the doses used in the present study are 10 times higher than those recommended by the US-OSHA as permissible exposure limits [18, 20]. On the other side, the doses selected were in the range of those used in other genotoxicity studies in order to make them comparable (Table 1).

### 2.5. Coexposure Treatments

The exposure regime was based on protocols for the rodent micronucleus assay [32, 33] and for the induction of xenobiotic metabolism enzymes [34]. Three groups of rats were used to test the effects of the mixture of VOCs. Three doses of a mixture of CLF/DCM/TOL (dissolved in corn oil) were intraperitoneally (i.p.) administered to the animals, a single dose for one day or one dose per day over 3 days. The doses to be used were set according to the results in the pilot study, so that the high dose, intermediate dose (mid-dose), and low dose each represent the 10, 5, and 2.5 percent of the LD50 for each compound in the mixture, respectively (Table 2, where the high dose corresponds to the doses used in the pilot study). The control group was given corn oil only.

Additionally, four groups of rats were treated with different chemicals that served as positive controls: trans-Stilbene oxide (tSBO) (2 mmol/kg, i.p., xenobiotic metabolism inducer), carbon tetrachloride (CCl_4_) (9.75 mmol/kg, i.p., oxidative stress damage), benzene (BEN) (12.8 mmol/kg, p.o., BM genotoxic damage), and diethylnitrosamine (DEN) (0.97 mmol/kg, i.p., hepatic genotoxic damage). The number of animals used for each treatment is presented in Table 2.

### 2.6. Sample Collection

The animals were euthanized by cervical dislocation 24 h after the last dose of the corresponding treatment. Livers were freshly excised and washed in cold 0.15 M KCl. Two small pieces (0.25 g approximately) of each liver were obtained to assess micronucleus in hepatocytes (MNHEP), proliferation (mitotic index), and glutathione (GSH/GSSG) levels. The fragment of liver designated for the evaluation of genotoxicity and proliferation was placed in 7 mL of 10% buffered formalin, and the fragment for quantification of GSH/GSSG was frozen in dry ice. Half of the liver from each animal was homogenized in 0.1 M phosphate buffer pH 7.0, with 0.1% Triton X-100, and centrifuged at 19,000 g for 10 min; the supernatant was used for the determination of the following antioxidant enzymes: superoxide dismutase (SOD), catalase (CAT), glutathione peroxidase (GPx), and glutathione reductase (GRed). The remaining liver was stored at −80°C until preparation of microsomal and cytosolic fractions (for no more than 2 weeks). Additionally, both femurs were removed to assess micronucleus in bone marrow polychromatic erythrocytes (MNPCE) and cytotoxicity (%PCE) in bone marrow.

### 2.7. Microsomal and Cytosolic Fractions

Microsomal and cytosolic fractions were prepared according to the procedure described by Guengerich [35] and Dávila-Borja et al. [36].

Protein concentrations in the microsomal and cytosolic fractions were determined using the protein assay dye reagent (Bio-Rad) according to supplier's instructions.

### 2.8. P-450 Determination

The cytochrome P-450 (P-450) content in the hepatic microsomal fraction was determined from the spectrum of the ferrous-carbon monoxide complex, using the molar extinction coefficient of 91 mM^−1^ cm^−1^ at 450/490 nm [35, 37]. Liver microsomes (0.5–1 mg/mL final concentration) were dissolved in 0.1 M potassium phosphate buffer pH 7.4, 1 mM EDTA, 20% glycerol (v/v), and 0.4% Triton X-100. A baseline from 400 to 500 nm was recorded and the sample cell was saturated with carbon monoxide, then both preparations were reduced with a few milligrams of Na_2_S_2_O_4_, and the spectral difference was recorded in the same wavelength range. P-450 content was expressed as nmol/mg protein.

### 2.9. CYP2E1 Specific Activity

CYP2E1 enzyme activity was determined by measuring the hydroxylation of 4-nitrophenol (4-NP) to 4-nitrocatechol (4-NCC) as described by Koop [38]. Briefly, the reaction mixtures contained 0.1 M potassium phosphate buffer and 1 mM ascorbic acid, pH 7, 0.1 mM 4-NP, hepatic microsomes (0.5–1 mg/mL final concentration), and 1 mM NADPH in a final volume of 1 mL. The reactions were initiated with NADPH after preincubation for 5 min at 37°C and were terminated with 0.2 mL of 1.5 M perchloric acid after 10 min of incubation at 37°C. The precipitated proteins were removed by centrifugation at 4,400 rpm for 5 min and supernatants were mixed with 0.1 mL of 10 N NaOH for the measurement of 4-NCC at 510 nm. The activity was expressed as nmol/min/nmol P-450.

### 2.10. GST Activity

Total GST activity was measured using the method described by Habig and Jakoby [39]. Briefly, the reaction mixture contained 50 mM potassium phosphate buffer pH 6.5, 0.05 mM GSH, 0.125 mM DNCB (2,4-dinitrochlorobenzene), and cytosol (0.03–0.05 mg/mL final concentration) in a final volume of 1 mL. The reactions were initiated with the cytosolic protein addition and the absorbance was recorded for 3 min at 340 nm. Enzyme activity was calculated using the molar extinction coefficient of the DNCB-GSH conjugate of 9.6 mM^−1^ cm^−1^. The activity was expressed as nmol/min/mg protein.

### 2.11. GSTT1 Activity

The glutathione-*S*-transferase T1 (GSTT1) hepatic activity was determined following the formation of formaldehyde from DCM [40]. Briefly, the reaction mixture contained 0.1 M TRIS/HCl pH 7.4, 10 mM GSH, cytosol (0.33 mg/mL final concentration), and 8 *μ*L DCM in a final volume of 3 mL. The reactions were initiated with DCM after preincubation for 5 min at 37°C and were terminated with 0.3 mL of a 50% aqueous trichloroacetic acid solution after 5, 10, and 20 min. The precipitated proteins were removed by centrifugation at 14,000 rpm for 2 min and 0.5 mL of the supernatant was mixed with 0.5 mL of Nash reagent (2 M ammonium acetate, 20 mM acetyl-acetone, and 50 mM acetic acid) and incubated at 42°C. After 30 min the absorption at 412 nm was measured and enzyme activity was calculated using the molar extinction coefficient of the DNCB-GSH conjugate of 8 mM^−1^ cm^−1^. The activity was expressed as nmol/min/mg protein.

### 2.12. GSH/GSSG Levels

Frozen liver samples were homogenized in 5 mL of 5-sulfosalicylic acid/g of tissue, using sonication (30 sec, 4.5 intensity, 4°C). The homogenates were centrifuged at 15,000 ×g for 3 min at room temperature and the acid supernatants were recovered.

Total glutathione was quantified in the acid supernatants using the enzymatic recycling assay of Anderson [41]. Briefly, the reaction mixture contained 143 mM sodium phosphate and 6.3 mM EDTA pH 7.5, 0.21 mM NADPH, 0.6 mM DTNB (Ellman's reagent), 1 *μ*L of acid supernatant, and 0.5 U of GRed in a final volume of 1 mL. The reactions were initiated with GRed after preincubation for 10 min at 37°C and absorbance at 412 nm was recorded for 3 min. The reaction rate (ΔAbs/min) was converted to nmol of GSH, using a standard curve of known amounts of GSH.

Quantification of oxidized glutathione (GSSG) was performed by derivatization of the reduced glutathione (GSH) present in the sample with 2-vinylpyridine prior to the enzymatic recycling assay, thus preventing GSH from participating in the reaction. Derivatization reaction contained 300 *μ*L of acid supernatants, 6 *μ*L of 2-vinylpyridine, and sufficient triethanolamine to bring the pH in the range of 6-7; reactions were incubated at room temperature for at least 60 min. The enzymatic reaction was in the same conditions as for the quantification of total GSH, except that the volume of the derivatized sample was 50 *μ*L.

The amount of GSH in the sample was calculated by subtracting the amount of GSSG from the amount of total glutathione. The results were expressed in nmol GSH or GSSG/g liver.

### 2.13. TBARS

TBARS were quantified using the method of Janero and Burghardt [42] as a surrogate for the estimation of malondialdehyde (MDA) content. Briefly, 1 g of each liver was homogenized with 500 *μ*L of a solution of 0.1 M butylated hydroxytoluene (dissolved in methanol/phosphate buffer 1 : 1). The homogenates were centrifuged at 3,000 g for 10 min and supernatants were recovered. Derivatization reaction contained 200 *μ*L of supernatants and 1 mL of a solution of thiobarbituric acid (26 mM TBA, 0.2 M HCl, 6.66% TCA, and 1 mM deferoxamine mesylate); reaction mixtures were heated in boiling water for 10 min. Reactions were cooled and 1 mL of n-butanol/pyridine (15 : 1) was added. After centrifugation at 1,200 g for 10 min, supernatants were recovered and their absorbance at 532 nm was recorded. Malondialdehyde (MDA) in whole tissue homogenates was measured using the extinction coefficient of 1.56 × 10^5^ M^−1^ cm^−1^, corresponding to the complex MDA-(TBA)_2_ and the results were expressed as nmol TBARS/mg protein.

### 2.14. CAT Assay

CAT activity was determined following the enzymatic decomposition of H_2_O_2_ [43]. Briefly, the reaction mixture contained 10 mM potassium phosphate pH 7.4, 5 *μ*L of homogenate dilution (1 : 40), and 30 mM H_2_O_2_ in a final volume of 725 *μ*L. The reactions were initiated with the addition of the sample and the absorbance at 240 nm was recorded for 30 seg. Under the conditions described, the decomposition of H_2_O_2_ by CAT contained in the samples follows a first-order kinetics as given by the equation *k* = 2.3/Δ*t*log⁡(*A*_1_/*A*_2_), where *k* is the first-order reaction rate constant, Δ*t*(*t*_2_ − *t*_1_) is the measured time interval, and *A*_1_/*A*_2_ is the absorbance at *t*_1_ and *t*_2_, respectively. The results were expressed in k/mg protein.

### 2.15. SOD Assay

SOD activity was measured by a competitive inhibition assay using xanthine-xanthine oxidase system to reduce nitroblue tetrazolium (NBT) [44]. The reaction mixture contained 160 *μ*L of 0.122 mM EDTA, 30.6 *μ*M NBT, 0.122 mM xanthine, 0.006% bovine serum albumin, and 49 mM Na_2_CO_3_, mixed with 33 *μ*L of liver homogenate (1 : 50 dilution) and 30 *μ*L of a xanthine oxidase solution to get a final concentration of 2.5 U/L; this mixture was incubated at room temperature for 30 min. The reaction was stopped with 66 *μ*L of 0.8 mM cupric chloride and the optical density was read at 560 nm. The 100% of NBT reduction was obtained in a tube in which the sample was replaced by distilled water. The amount of protein that inhibited 50% of NBT reduction was defined as one unit of SOD activity. Results were expressed as U/mg protein.

### 2.16. GPx Assay

GPx activity was assayed by a coupled reaction with glutathione reductase (GRed) [45]. The reaction mixture consisted of 50 mM potassium phosphate solution pH 7.0, 1 mM EDTA, 1 mM sodium azide, 0.2 mM NADPH, 25 U/mL of GRed, and 1 mM GSH at 25°C. 100 *μ*L of liver homogenate diluted 1 : 10 was added to 800 *μ*L of the reaction mixture and allowed to incubate for 5 min at room temperature before initiating the reaction by the addition of 32 *μ*L of 2.5 mM H_2_O_2_ solution. Absorbance at 340 nm was recorded for 3 min and the activity was calculated from the slope of these curves as *μ*moles of NADPH oxidized per min taking into account that the millimolar absorption coefficient for NADPH is 6.22 mM^−1^ cm^−1^. Blank reactions with homogenates replaced by distilled water were subtracted from each assay. One unit of GPx was defined as the amount of enzyme that oxidizes 1 *μ*mol of NADPH/min. The results were expressed as U/mg protein.

### 2.17. GRed Assay

GRed activity was spectrophotometrically assayed using GSSG as substrate and measuring the disappearance of NADPH at 340 nm [46]. The reaction mixture consisted of 0.1 M potassium phosphate and 0.5 mM EDTA, pH 7.6, 1.25 mM NADPH, and 0.5 mM GSSG at 25°C. 25 *μ*L of diluted homogenate (1 : 5) was added to 475 *μ*L of reaction mixture. Absorbance at 340 nm was recorded for 3 min and the activity was calculated from the slope of the curves as *μ*moles of NADPH oxidized per min. One unit of GRed was defined as the amount of enzyme that oxidizes 1 *μ*mol of NADPH/min. The results were expressed as U/mg protein.

### 2.18. Bone Marrow Micronucleus Test

Evaluation of MNPCE was performed according to the procedure of Romagna and Staniforth [47]. BM of a femur was prepared with newborn calf serum (Invitrogen Co.), 25 mM EDTA (3 mL for both femora). Cell suspension was carefully dropped into the center of a cellulose column (Sigma cell type 50 and *α*-cellulose) and 25 mL of Hank's balanced salt solution (HBSS) was added to the column surface. The eluate containing the erythrocytic cells was washed twice in 20 mL of HBSS and centrifuged at 2,200 rpm for 10 min. Finally the pellet was homogenized in the minimum volume of HBSS and the slides were prepared using 3 *μ*L of the pellet.

### 2.19. %PCE

Two smears were made per animal and slides were stained with undiluted Wright-Giemsa (Sigma). A total of 2,000 polychromatic erythrocytes (PCE) from each rat were evaluated for the micronucleus frequency. Additionally, BM cytotoxicity was evaluated by recording the %PCE present in 2,000 erythrocytes per animal.

### 2.20. Micronucleus in Hepatocytes (MNHEP)

Formalin-fixed tissue was used according to the method of Parton and Garriott [33] with modifications. Pieces of liver previously fixed in 10% buffered formalin for at least seven days were placed individually into flasks containing 7 mL of 12 N KOH and agitated on a shaker for ~16 hr at room temperature. The liver pieces were carefully placed in a brass cloth (Tyler equivalent 100 mesh size) and rinsed with distilled water. Hepatocytes were dissociated through the cloth using a Teflon pestle and collected in a 50 mL centrifuge tube. The cell suspension was centrifuged at 400 rpm for 10 min, and the water carefully aspirated. The pellet was resuspended in 50 mL distilled water and centrifuged at 400 rpm for 10 min; this step was repeated. After the third centrifugation, the pellet was resuspended in 3 mL of a methanol-acetic acid (3 : 1) fixative solution and stored at 4°C until slides were prepared.

Two smears were made per animal and slides were stained with undiluted Wright-Giemsa. A total of 2,000 hepatocytes with good morphology from each rat were evaluated for the micronucleus frequency.

### 2.21. Mitotic Index

In the same slides used for micronucleus determination, one thousand hepatocytes were counted per animal, enumerating the amount of mitotic figures. The mitotic index was calculated as the number of mitosis observed/one thousand cells observed.

### 2.22. Statistical Analysis

All experiments described were done by triplicate and data were captured and analyzed using Stata 7.0 software. Values were expressed as mean ± s.d. and group comparisons were assessed using Kruskal-Wallis test. Pearson correlations were explored among data after one-day treatment or three-day treatments, and linear regression was used to analyze the correlations found. Differences between negative and positive controls were calculated by the Student's *t*-test. Significance was established at a level of *P* ≤ 0.05.

## 3. Results

Effects on the parameters studied were clearly different between one-day and three-day treatments (Table 3).

### 3.1. One-Day Treatments

**Figure 1:**
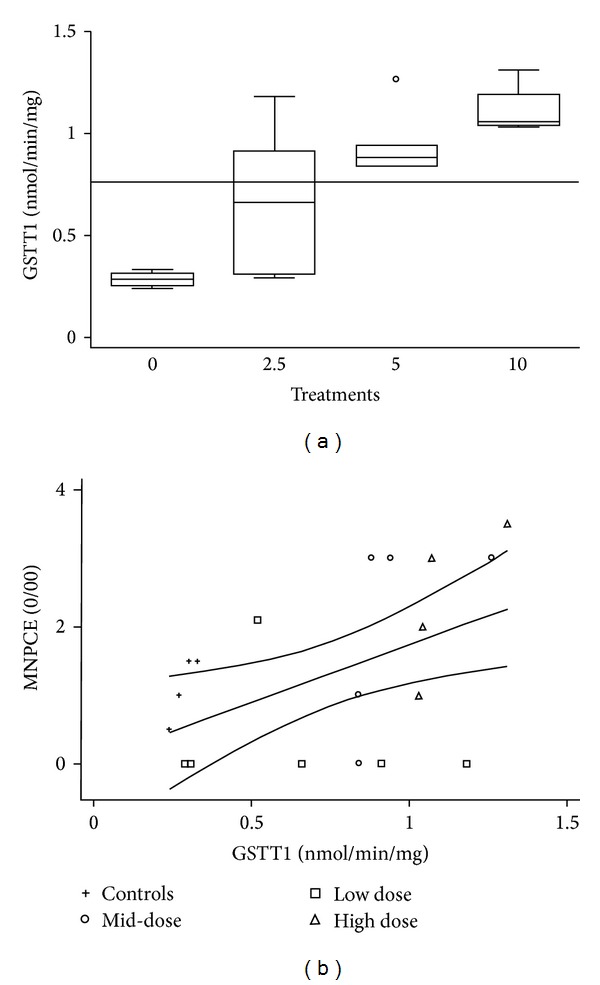
One-day treatment. (a) GSTT1 metabolic enzyme showed increased activity with the dose. Boxes represent the first and the third quartile and the median value. The line behind the bars represents the overall mean for this enzyme activity. (b) GSTT1 induced activity correlated with MNPCE, *R* ^2^ = 0.24, *P* = 0.037.

The simultaneous exposure to the three compounds at different concentrations after one-day treatment resulted in the increased activity of metabolic enzyme GSTT1 with each treatment (Pearson coefficient = 0.76; *P* = 0.0001, Table 4 and Figure 1(a)), whereas activity of other GST enzymes was inhibited with the low and high doses (Table 3). CYP2E1 showed increased activity only with the high dose (Pearson coefficient 0.61; *P* = 0.004, Table 4), even though the total P-450 hepatic content did not change (Table 3). The antioxidant response, measured through the activity of enzymes SOD, GPx, and GRed, did not show a significant change (Table 3). The ratio of GSH over GSSG was not altered with any dose, and no significant change in TBARS was observed (Table 3).

Micronuclei in PCE showed increased frequencies with the higher doses with a maximal increase of 2.7-fold and a Pearson correlation with the treatments with a coefficient value of 0.57 and *P* = 0.007 (Tables 3 and 4), whereas %PCE showed no significant change. Interestingly, increased MNPCE were correlated with GSTT1 activity in the liver; Pearson coefficient = 0.49; *P* = 0.04.  Figure 1(b) shows the linear regression of this result.

Increased MNHEP were observed in the liver related with the dose with a maximal increase of 11-fold which was close to significance (Pearson coefficient = 0.44; *P* = 0.054, Tables 3 and 4). However, a reduction of proliferation was observed related with the dose (Pearson coefficient = −0.48; *P* = 0.04, Table 4).

A summary of the correlations found with this regime of treatment is presented in Table 4.

### 3.2. Three-Day Treatments

Contrary to what was observed in the single-day treatment, three-day treatments with the mixture produced significant responses in the metabolic enzymes at the low dose. Total P-450 were induced in the low dose and then a significant reduction with the dose was observed (Kruskal-Wallis chi value 18.7, *P* = 0.0003), and CYP2E1 activity was significantly increased at low and mid-doses in contrast with the one-day treatment (Kruskal-Wallis chi value 22.2, *P* = 0.0001) (Table 3). GSTs were not altered with this regime of treatment; however, GSTT1, which is involved in the metabolism of DCM, showed a significant 1.75-fold induction in the low dose (Kruskal-Wallis, chi value 13.5, *P* = 0.004), not as high as 2.4-fold as was induced with the same dose with the one-day treatment (Table 3); compared to this regime it would appear that the enzyme was affected in its activity by the treatments for three days.

**Figure 2:**
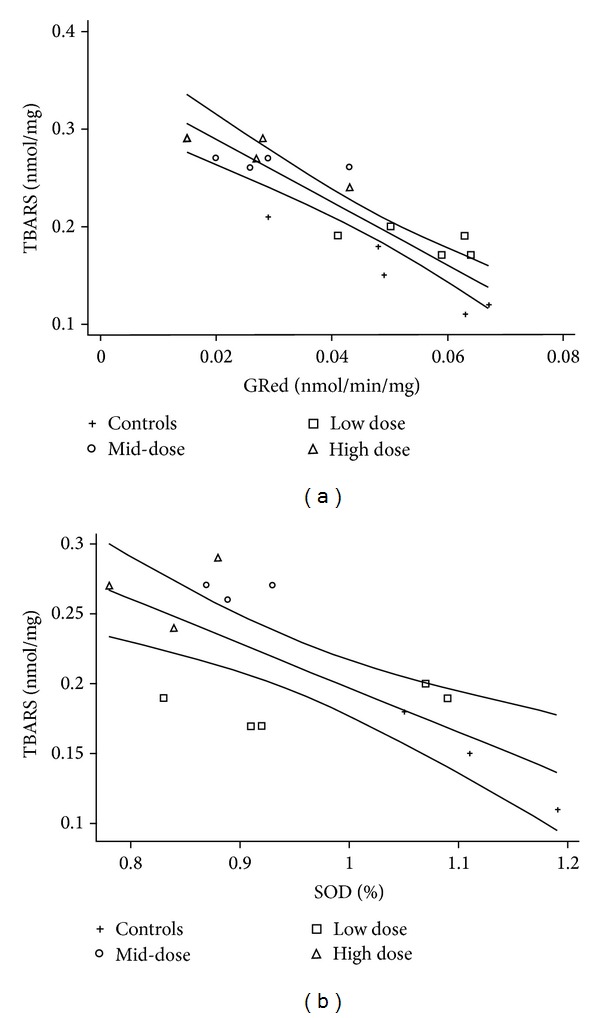
Three-day treatment. TBARS were increased in a dose-related manner, probably as the result of the reduced activity of antioxidant enzymes like GRed which showed an inverse correlation with it (a), *R* ^2^ = 0.76, *P* = 0.00001, and SOD which also showed an inverse correlation (b), *R* ^2^ = 0.52, *P* = 0.004.

Antioxidant enzyme GPx showed significantly decreased activity with the low and high dose (Kruskal-Wallis chi value 11.5, *P* = 0.007), GRed showed a significant reduction with the dose (Pearson coefficient = −0.61; *P* = 0.01), and SOD also exhibited a decreased activity related with the treatments (Pearson coefficient = −0.75; *P* = 0.002), while CAT showed nonsignificant reductions with treatments. These findings were consistent with the significant reduction of the GSH/GSSG ratio at the high dose (Pearson coefficient = −0.51; *P* = 0.002, Table 5) and it was correlated with the induced activity of metabolic enzymes CYP2E1 and GSTT1 with identical Pearson coefficient = 0.55, and *P* = 0.003. Oxidative stress was observed under this regime, producing increased levels of TBARS with each dose (Pearson coefficient = 0.82; *P* = 0.00001, Table 3) which significantly correlated with GRed activity (Pearson coefficient = −0.87; *P* = 0.00001) and SOD activity (Pearson coefficient = −0.72; *P* = 0.004); Figure 2 shows the linear regressions. A summary of the correlations found with this regime of treatment is presented in Table 5.

The oxidative stress was not reflected in micronucleus production in the BM, whereas in the liver MNHEP were increased in the high dose (3-fold), although at a lower level than with the one-day treatment. A greater variability in %PCE was observed in the BM with significant reductions at the low and high doses (Kruskal-Wallis, chi value 8.6, *P* = 0.03, Table 3), whereas in the liver, the proliferation was generally lower than with the one-day treatment and the highest dose produced a significant cytotoxic effect (Kruskal-Wallis, chi value 8.2, *P* = 0.03, Table 3).

## 4. Discussion

In order to gain insight into the relationship of metabolism, oxidative stress, and micronucleus production related with the coexposure to CLF, TOL, and DCM, our study considered two different regimes of exposure in a rat model: a single-day treatment and a three-day treatment (one dose/day). The two regimes produced a different pattern of response (Table 3) in all the parameters.

### 4.1. One-Day Treatments

**Figure 3:**
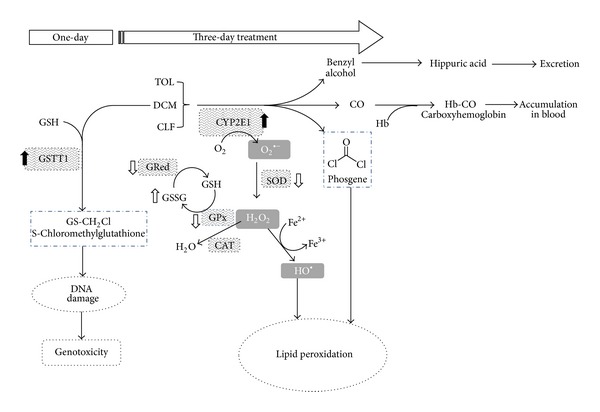
Diagram representing how the combined metabolism of the three compounds could induce the responses observed. After one-day treatments, GSTT1 and CYP2E1 induction could contribute to the generation of metabolites and ROS to produce increased MNPCE. After three-day treatments, still induced CYP2E1 and reduced activity of antioxidant enzymes (empty arrows) lead to the accumulation of H_2_O_2_ and the superoxide anion, damaging membranes and allowing an oxidative stress.

No change in the antioxidant response was observed under the single-day treatment and oxidative stress biomarkers such as TBARS and the GSH/GSSG ratio were not altered. Phase I and phase II enzymatic activity, in turn, exhibited induction; that is, CYP2E1 and GSTT1 activities were induced (Table 3). CYP2E1 is involved in the biotransformation of the three compounds tested, whereas GSTT1 participates in the bioactivation of DCM, producing a metabolite suspected to be the precursor of formaldehyde, a known genotoxic carcinogen [1] (Figure 3). GSTT1 in this instance would not be acting as a phase II conjugating enzyme but more as an activating enzyme as has been described in the metabolism of DCM.

MNPCE showed an increase related with the dose and interestingly, they were correlated with GSTT1 activity. This was the only parameter measured in the liver that showed a correlation with BM MNPCE, which could be explained in two possible ways: (1) The exposure to the mixture of pollutants could induce GSTT1 activity in the erythroid line, this process could increase bioactivation of DCM on the bone marrow, leading to genotoxicity [30, 48, 49], or (2) The bioactivation of DCM in liver produces a reactive metabolite that is transported by the bloodstream into the bone marrow, causing the genotoxic damage. It is known that the modulation of GSTT1 activity directly affects the metabolism of DCM, so that if there is an increase in the GSTT1 activity the DCM metabolism is higher and vice versa [50, 51]; therefore it is reasonable to think that the relation between GSTT1 and MNPCE could be linked to the metabolism of DCM.

### 4.2. Three-Day Treatments

The three-day regime exerted a more intensive oxidative effect than the single-day treatment, reducing the activity of the antioxidant enzyme GPx (which reduces H_2_O_2_ into water), inducing CYP2E1 (whose activity generates H_2_O_2_ and superoxide anion), and producing damage to lipids (TBARS) in all doses, which was inversely correlated with GRed (which has the function of recovering GSH from GSSG and making it available to protect the cell from oxidation) and SOD (which conjugates superoxide anion) activities. The ratio GSH/GSSG was first induced and then decreased with increasing doses in a similar manner as CYP2E1; these two parameters showed a significant correlation and a similar correlation was found with induced GSTT1 (Table 5).

Previous studies showed little evidence of oxidative stress produced by the individual compounds when administered* in vivo* at doses even higher than the ones used in the present study [9, 24, 27, 28, 52] (Table 1) and we did not find changes in GSH levels with any of the individual compounds administered at the high dose for three days in the pilot study either (Table 1).

Our results are comparable to those obtained by Bird et al. [10] with benzene and TOL where MNPCE produced by benzene were increased in a coexposure to TOL, but they decreased upon GSH depletion; in the single-day treatment we observed increased MNPCE but not so after three-day exposure where GSH/GSSG ratio was significantly reduced, correlating with a lower activity of CYP2E1 and GSTT1 (Table 5). This might have to do with a reduced formation of toxic metabolites, like S-chloromethylglutathione (which is suspected of producing sister chromatid exchanges [53]), and for that reason no significant MN induction was observed (Figure 3).

P-450 levels under the three-day treatment were significantly induced in the low dose, but then they decreased with the dose, being significantly reduced only in the high-dose treatment (Table 3).

CYP2E1 activity, in turn, was significantly increased at the low and mid-doses. When individually administered in the pilot study, CLF and TOL also produced an induction (Table 1). Similar induction was found by González-Jasso et al. [29] with TOL alone in 5 mmol/kg doses and by Pathiratne et al. [52] with a higher dose (20 mmol/kg) of TOL (Table 1). CYP2E1 is the P-450 isoform involved in these VOCs metabolism and its increased activity was expected. Induction of this enzyme is part of a mechanism of adaptive response to chemicals and its deregulation may have important toxicological consequences; for example, the induction of CYP2E1 has been associated with an increment of ROS production in the liver and this process is thought to contribute to alcohol-dependent liver injury [54], as well as the induction of CYP2E1 by solvents, prior to the exposure to DCM, increased blood carboxyhemoglobin levels in rats [55, 56] due to CO produced in the metabolism of DCM. Metabolism by CYP2E1 has also been described in the toxic pathway of chloroform [57] to produce phosgene adducts in the amino terminus of human histone H2B [58], probably mimicking the acetylation of the histone with consequences for gene expression. Figure 3 represents how the results found could be explained based on what is known about the metabolism of these compounds.

In relation with the type of interaction of the three compounds, it depends on the doses used and on the biomarkers taken as a reference. Based on previous studies (Table 1), we can say that the response by the xenobiotic metabolizing enzymes was similar to the response obtained in exposures to individual compounds after three days; however, in relation to the oxidative response, the mixture produced an oxidative stress that was never observed with individual compounds at the same doses; hence, the coexposure resulted in synergistic effects affecting the antioxidant response of the organism.

### 4.3. Oxidative Stress and Micronuclei

Given that an oxidative stress was induced with the treatments, an increase in MN was expected either in the BM or in the liver. Individual compounds had been analyzed for their genotoxicity and the results were inconsistent, indicating some clastogenic activity for the three compounds but not in every test [25, 26, 31, 53, 59] (Table 1). In our pilot study no significant genotoxic effect with any of the individual compounds in the bone marrow was found with a three-day treatment either (Table 1). With the coexposure after three days, MNHEP did not increase, at the same time that the mitotic index was significantly reduced with the high treatment (9-fold). In visual inspections of the liver of animals who received three treatments with the high dose, fatty scars were observed. Increased MN with respect to the one-day treatment was expected in the BM as well, but it was not found and a trend at reduced %PCE was found, being significant at the low and high doses (Kruskal-Wallis test, *P* = 0.04). %PCE in the BM can be used to indicate BM toxicity; when replacement of existing PCEs from the erythroblast pool is depressed, the %PCE will decrease [60].

### 4.4. MNHEP

Micronuclei in hepatocytes had not been reported before for these compounds. They are weakly genotoxic to the BM and according to our results their genotoxicity in a coexposure would depend on the induction of metabolic enzymes like GSTT1 and hence to the production of genotoxic metabolites. The observed effect on hepatocytes was 5- to 11-fold higher than in controls after only one-day treatment; however, proliferation in the liver was reduced at the same time. Hepatic proliferation is considerably lower than in the BM and the proliferation of cells is necessary for the formation of MN, and even though young rats were used and an increase with the dose was found (Table 4), only 46% of the animals treated with any dose of the mixture showed MNHEP; an interesting finding was that, among these animals who showed an increase, it was significantly correlated with CYP2E1 induced activity (*P* < 0.04, data not shown) in a similar effect as that of the metabolic polymorphisms in humans. Conversely 75% of the animals were responsive to DEN, the positive control; it did not affect the proportion of evaluatable cells and even induced proliferation (Table 3). On the other hand, three days of treatment were too oxidative, but no increase in genotoxicity was observed.

### 4.5. Differences between One-Day and Three-Day Treatments

The toxic effects of xenobiotics depend on the dose and on the time of exposure. In the present study, three doses of the mixture under two types of xenobiotic exposure regimen (single and repeated doses) were tested. This type of experimental design was useful for understanding the toxicological behavior of the mixture of VOCs in different scenarios. The single exposure regime was used to evaluate the first responses of the organism when exposed to a mixture of VOCs, while the regimen of repeated doses was used to assess the accumulation of damage.

The single dose protocol revealed that the biomarkers of oxidative stress and cytotoxicity were maintained at normal levels, whereas the biomarkers of xenobiotic metabolism and genotoxicity increased with the dose. These results can be interpreted as follows: with a single dose, defense systems are not exceeded and are able to maintain cell homeostasis; however the organism is able to sense the presence of xenobiotics and activates the metabolism to accelerate detoxification; the increase in metabolism also increases the levels of reactive metabolites and biomolecular damage (micronuclei) is more probable.

With the repeated dose protocol, biomarkers of oxidative stress, membrane damage, cytotoxicity, and xenobiotic metabolism were increased with the dose, whereas genotoxicity was decreased. This result indicates that damage to macromolecules accumulated and the defense system was completely exceeded, leading to cell injury or death. Since the formation of micronuclei depends on cell proliferation, cell arrest or cytotoxicity would explain the decrease in micronuclei frequency. These results are in contrast to what was found with individual compounds in the pilot study which did not induce genotoxicity, cytotoxicity, or oxidative stress; however, in a coexposure like this, the outcome was synergistic and even overpassed the antioxidant defense of the organism causing visible liver damage comparable to what has been described about alcohol-dependent liver injury [54].

In summary, the use of two exposure regimes allowed us to propose scenarios where the cellular response is sufficient to maintain the viability even if sustaining a genotoxic effect that could translate in subtle alterations on the long term, or when the response is completely exceeded, compromising cellular integrity that could lead to tissue illness in a short period of time.

## 5. Conclusions

The coexposure to CLF, DCM, and TOL induced the activity of metabolism enzyme GSTT1 and it was correlated with the micronucleus frequency in the bone marrow, after only one treatment. Even though the micronuclei induction was not as high as it is with benzene or other well-established clastogenic agents, these lesions could be of relevance in a prolonged exposure regime or in a combined exposure with a clastogenic agent which is possible in a polluted environment scenario. At the same time ROS could have been produced by the induced activity of CYP2E1, generating genotoxicity, but in levels not affecting the activity of antioxidant enzymes or GSH levels, opening the possibility that a lower and sustained exposure over time could produce significant chromosomal damage in both tissues. Future experiments would help dilucidate this matter.

Sustained exposure for three days under this regime led to oxidative stress at all doses without affecting the survival of the animals but producing fat liver.

## Figures and Tables

**Table 1:** Acute toxic effects in rodent i.p. exposed to dichloromethane (DCM), chloroform (CLF) or toluene (TOL).

VOC	Dose (mmol/kg)	P-450	CYP2E1	Lipid peroxidation	GSH	MNPCE	CA	Reference
DCM	2.5	0	0		0	0		Pilot study
CLF	2.6	0	+0.48		0	0		Pilot study
TOL	8	−0.48	+0.50		0	0		Pilot study

DCM	1.2–2.4	0	0					[23]
	4.8–9.5	+0.4–0.50	+0.35					[23]
	6			0				[24]
	5–20					0		[25]
	1.2–23.5						0	[26]

CLF	0.1				0			[27]
	1.3	0	0		0			[28]
	1.7				−0.07			[27]
	2.0–8.0					0		[25]
	0.01						+3.75	[21]
	0.1–1.0						+7.75	[21]

TOL	5	0	+1.16					[29]
	5.4			0				[9]
	10.8			+0.30				[9]
	16.2			+0.17				[9]
	20				0			[30]
	1.2					0	0	[31]
	2.4					+0.32	0	[31]
	4.7					0	+2.71	[31]

Data represent significant fold increases (+) or decreases (−) with respect to control animals; zero means no change.

Cytochrome P450 (P-450), cytochrome 2E1 (CYP2E1), lipid peroxidation and glutathione (GSH) were determined in liver, micronuclei (MNPCE) and chromosomal aberrations (CA) were determined in bone marrow.

**Table 2:** Doses administered per treatment.

Group of treatment	Doses	*N*
Neg. control (corn oil)*	125 *μ*L/kg b.w.	5
	TOL/DCM/CLF^*⋆*^	
Low-mix*	2.0/0.6/0.65	5
Mid-mix*	4.0/1.2/1.3	5
High-mix*	8.0/2.5/2.6	5

Positive controls^*⋆*^		
tSBO	2.0	3
CCl_4_	10.0	3
BEN	12.0	3
DEN	1.0	3

*These were the daily doses administered for one day or three days of the mixture.

^*⋆*^(mmol/kg b.w.).

BEN and DEN were administered for two days; CCl_4_ and tSBO, for three days.

*N* refers to animals used in either regime of treatment.

**Table:** (a)

Treatment	Phase I and phase II xenobiotic metabolism				
	*n*	[CYP] (nmol/mg)	CYP2E1 (nmol/min/mg)**	GST (mmol/min/mg)	GSTT1 (nmol/min/mg)**
CT	5	0.79 ± 0.11	2.23 ± 0.34	0.47 ± 0.10	0.28 ± 0.04
Low Dose	5	0.76 ± 0.08	1.94 ± 0.24	0.27 ± 0.08	0.67 ± 0.38
Mid Dose	5	0.88 ± 0.13	2.23 ± 0.28	0.44 ± 0.06	0.93 ± 0.19
High Dose	5	0.74 ± 0.13	3.09 ± 0.77	0.34 ± 0.05	1.11 ± 0.13

Treatment	Antioxidant enzymes				
	*n*	SOD^†^	GPx (U/mg)	Gred (U/mg)	

CT	5	1 ± 0.12	0.18 ± 0.05	0.072 ± 0.006	
Low Dose	5	0.85 ± 0.18	0.14 ± 0.01	0.068 ± 0.007	
Mid Dose	5	0.96 ± 0.21	0.18 ± 0.02	0.073 ± 0.011	
High Dose	5	0.91 ± 0.14	0.18 ± 0.03	0.073 ± 0.001	

Treatment	Oxidative stress				
	*n*	[GSH] (mmol/g)	[GSSG] (mmol/g)	[GSH]/[GSSG]	TBARS (nmol/mg)

CT	5	2.97 ± 0.95	0.10 ± 0.05	27.49 ± 8.53	0.18 ± 0.05
Low Dose	5	4.34 ± 0.92	0.13 ± 0.03	35.06 ± 10.70	0.28 ± 0.10
Mid Dose	5	2.44 ± 0.8	0.10 ± 0.07	33.02 ± 15.71	0.21 ± 0.05
High Dose	5	3.05 ± 1.13	0.10 ± 0.06	28.61 ± 21.14	0.17 ± 0.03

Treatment	Genotoxicity and proliferation				
	*n*	MNPCE (‰)**	%PCE	MNHEP/1000	Metaphase (‰)

CT	5	1.20 ± 0.44	42.55 ± 8.43	0.1 ± 0.17	5.63 ± 3.70
Low Dose	5	0.00 ± 0.00	31.08 ± 12.28	0.30 ± 0.44	3.90 ± 3.27
Mid Dose	5	2.00 ± 1.41	43.64 ± 17.62	0.40 ± 0.65	2.00 ± 2.18
High Dose	5	3.20 ± 2.07	43.45 ± 7.87	0.70 ± 1.10	2.20 ± 1.10
Ben/DEN	3	10.75 ± 4.21*	49.76 ± 5.30	1.33 ± 1.41*	9.75 ± 3.10

**(b):** 

Treatment	Phase I and phase II xenobiotic metabolism				
	*n*	CYP (nmol/mg)**	CYP2E1 (nmol/min/mg)**	GST (mmol/min/mg)	GSTT1 (nmol/min/mg)**

CT	5	0.54 ± 0.11	1.06 ± 0.29	0.47 ± 0.06	0.33 ± 0.10
Low Dose	5	0.72 ± 0.09	1.64 ± 0.22	0.46 ± 0.08	0.58 ± 0.13
Mid Dose	5	0.60 ± 0.10	1.96 ± 0.32	0.51 ± 0.07	0.38 ± 0.16
High Dose	5	0.41 ± 0.08	1.06 ± 0.21	0.50 ± 0.07	0.34 ± 0.11
tSBO	3	0.85 ± 0.07*	2.36 ± 0.14	0.53 ± 0.06	0.74 ± 0.18*

Treatment	Antioxidant enzymes				
	*n*	SOD^†∗∗^	GPx (U/mg)**	Gred (U/mg)**	CAT (k/mg)

CT	5	1.11 ± 0.07	0.13 ± 0.01	0.05 ± 0.014	0.51 ± 0.12
Low Dose	5	0.96 ± 0.11	0.08 ± 0.01	0.05 ± 0.010	0.50 ± 0.15
Mid Dose	5	0.89 ± 0.03	0.11 ± 0.01	0.03 ± 0.007	0.42 ± 0.05
High Dose	5	0.83 ± 0.05	0.08 ± 0.02	0.03 ± 0.012	0.39 ± 0.06
CCl_4_	3	0.71 ± 0.06*	0.08 ± 0.02*	0.02 ± 0.005*	0.3 ± 0.13*

Treatment	Oxidative stress				
	*n*	[GSH] (mmol/g)**	[GSSG] (mmol/g)**	[GSH]/[GSSG]**	TBARS (nmol/mg)**

CT	5	6.79 ± 4.02	0.15 ± 0.07	47.30 ± 15.08	0.15 ± 0.04
Low Dose	5	15.57 ± 1.63	0.18 ± 0.08	95.21 ± 33.83	0.19 ± 0.01
Mid Dose	5	7.63 ± 0.64	0.13 ± 0.03	60.91 ± 11.21	0.27 ± 0.005
High Dose	5	7.02 ± 1.15	0.53 ± 0.36	16.05 ± 5.42	0.27 ± 0.02
CCl_4_	3	10.38 ± 3.6	0.52 ± 0.24*	20.53 ± 2.95*	0.19 ± 0.02*

Treatment	Genotoxicity and proliferation				
	*n*	MNPCE (‰)	%PCE**	MNHEP/1000	Metaphase (‰)**

CT	5	1.88 ± 1.27	53.58 ± 5.80	0.1 ± 0.17	2.30 ± 2.52
Low Dose	5	1.13 ± 0.64	40.23 ± 12.87	0.19 ± 0.25	2.13 ± 1.25
Mid Dose	5	0.75 ± 1.07	52.91 ± 2.80	0.06 ± 0.17	2.1 ± 2.7
High Dose	5	0.56 ± 0.50	43.75 ± 11.84	0.31 ± 0.45	0.25 ± 0.46
Ben/DEN	3	5.38 ± 3.12*	56.61 ± 2.39	1.33 ± 1.41*	9.75 ± 3.10*

Mean values plus standard deviations are presented for all the parameters.

*Positive controls significantly different from negative controls (Student *t*-test).

**Parameters where a difference due to the treatment was found at least in one dose. Kruskal-Wallis rank test, *P* ≤ 0.05. See text for details.

^†^Relative units.

**Table 4:** Correlations found between parameters after one-day treatments.

	Treatments	GSTT1 (nmol/min/mg)	TBARS (nmol/mg)
GSTT1 (nmol/min/mg)	0.76 **0.0002**		
MNPCE (‰)	0.57 **0.007**	0.49 **0.04**	
MNHEP (‰)	0.44 0.054*		
CYP2E1 (nmol/min/mg)	0.61 **0.004**	0.48 **0.04**	−0.48 **0.04**

Pearson coefficients.

*P* values are in bold.

*Close to significance.

**Table 5:** Correlations found between parameters after three-day treatments.

	Treatments	TBARS (nmol/mg)	GSTT1 (nmol/min/mg)	CYP2E1 (nmol/min/mg)	P-450 (nmol/mg)	CAT (k/mg)
GRed (U/mg)	−0.61 **0.01**	−0.87 **0.00001**				
P-450 (nmol/mg)	−0.48 **0.01**			0.62 **0.0004**		
GSH/GSSG (ratio)	−0.51 **0.002**		0.55 **0.003**	0.55 **0.003**	0.68 **0.00001**	
TBARS (nmol/mg)	0.82 **0.00001**					
SOD	−0.75 **0.002**	−0.72 **0.004**				0.56 **0.04**
GSTT1 (nmol/min/mg)				0.38 **0.03**		
GPx (activity)			−0.55 **0.02**			

Pearson coefficients.

P values are in bold.
